# “Because I’ve Got a Learning Disability, They Don’t Take Me Seriously:” Violence, Wellbeing, and Devaluing People With Learning Disabilities

**DOI:** 10.1177/0886260521990828

**Published:** 2021-02-02

**Authors:** Phillippa Wiseman, Nick Watson

**Affiliations:** 1 University of Glasgow, Scotland, UK

**Keywords:** bullying, hate crimes, violence exposure, community violence, learning disability, wellbeing, UK

## Abstract

For people with learning disabilities, targeted violence has become routinized. In this article, we seek to explore the impact pervasive victimization has on their experience of community and participation and, through this, their health and wellbeing. People with learning disabilities experience significant inequality in health and wellbeing compared to their non-disabled peers, and the role of violence and victimization remains mostly neglected. By drawing on in-depth qualitative interviews with people with learning disabilities, we argue that abuse, disrespect and devaluing profoundly erode wellbeing. The complex forms of violence experienced by people with learning disabilities are critical to understanding the significant inequalities in health and wellbeing experienced by people with learning disabilities. We focus on community and misrecognition to move the focus from one that examines causation towards one that uncovers the layers of invisibility, and the complex relations that structure experiences from the perspective of people with learning disabilities themselves. By doing this, we locate violence and victimization as health and wellbeing concerns and seek to add a more comprehensive and holistic understanding of the social determinants of health. For the inequalities that structure the lives of people with learning disabilities to be holistically understood, they must be reframed as an issue of social justice, and violence must be identified as a central contributor to these inequalities.

## Introduction

The violent victimization of people with learning disabilities is now well established; what is much less well understood is the impact persistent victimization has on the lives and wellbeing of people with learning disabilities. We will explore how violence shapes health and wellbeing, and the barriers it places on people with learning disabilities’ ability to participate in communities. People with learning disabilities often live isolated lives and feel excluded from their local communities (Power & Bartlett, 2018a, Hall & Bates, 2019). Despite its ubiquity, the role violence plays in this process has not been explored. Understanding violence against marginalized groups, and its impact, requires “that our analyses go beyond the patterning of crime rates” (Gadd & Corr, 2017, p. 69; Hydén, 2015) and prioritize the narratives of those who experience violence. In this article, we draw on people with learning disabilities’ experiences of violence in communities and its impact on their wellbeing to explore the relationship between violence, wellbeing, and belonging.

People with learning disabilities’ accounts of violence and victimization are essential to understand if we are to appreciate the factors that underpin the significant inequalities in health and wellbeing experienced by people with learning disabilities. People with learning disabilities live shorter lives; the life expectancy for women with learning disabilities is 18 years less than for women in the general population and for men with learning disabilities, 14 years shorter than the general population (O’Leary et al., 2018). They also experience significantly more physical and mental ill-health and have as many health conditions at age 20 years and over, as the rest of the population has by aged 50 years (O’Leary et al., 2018). Their wellbeing and mental health are poorer; adults with learning disabilities have significantly higher rates of a range of conditions, including schizophrenia, depression, and anxiety (Cooper et al., 2015).

There is increasing public and social awareness of the preventable deaths linked to poor health care of people with learning disabilities in the United Kingdom. For example, the death of 33-year-old Richard Handley from complications related to constipation (Down’s Syndrome Association, 2018)^1^1Richard Handley was a young man with Down’s Syndrome who died at age 33 in 2012 due to failures in health and social care. He died with 10 kg of feces in his bowel as a result of gross negligence on the part of the hospital monitoring his care. and the preventable death of 18-year-old Connor Sparrowhawk by neglect and mistreatment (to name only two; Ryan, 2017).^2^2Connor Sparrowhawk died by drowning in a bath in NHS care when he had an epileptic seizure despite health care professionals being informed that he should not bathe alone for this reason. There is, simultaneously, increasing reporting of the various forms of violence experienced by people with learning disabilities living in residential settings such as Assessment and Treatment Units (ATUs),^3^3Assessment and Treatment Units refers to secure treatment units for adults with learning disabilities in the United Kingdom. ATUs are typically supposed to be short-term placements for treatment and assessment while people with learning disabilities often remain detained in ATUs (away from their families) for undetermined lengths of time. For more information: https://www.challengingbehaviour.org.uk/information/information-sheets-and-dvds/assessmentandtreatmentunits.html care homes, and supported accommodation settings. The cruelty and abuse (Flynn, 2012) perpetrated at, private care homes, such as Winterbourne View in 2011 (Sin, 2014) and Whorlton Hall in 2019 (Iacobucci, 2019), exemplify the kind of everyday, endemic, hidden violence that people with learning disabilities are subject to. In the case of Winterbourne View, a considerable part of the abuse experienced by people with learning disabilities was the neglect of provision of health care or enforced and unnecessary drug prescribing (Flynn & Hollins, 2013). There have also been well documented and publicized cases of extreme violent victimization of people with learning disabilities in communities in the United Kingdom. The violent deaths of Brent Martin and Fiona Pilkington, for example, evidence what Sherry (2010, p. 88) refers to as “shockingly brutal” and “hyper-violent” and resulting from sustained victimization and negligence. Despite these events, there has been little change in people with learning disabilities’ experiences of violence in communities and residential care settings.

There are, according to Emerson and Hatton (2014), five broad causes of the health inequalities experienced by people with learning disabilities: the social determinants of health, including poverty, poor housing, and exclusion from community participation; impairment effects; communication barriers; personal health risks; and, poor access to health care. The impacts of the social determinants of health for people with learning disabilities has received limited attention. The influence of poverty, the impact of socioeconomic disadvantage, discrimination and exclusion, targeted violence, and the other “social gradients” that structure inequalities for people more generally have not been considered in detail in social or health research (Emerson & Hatton, 2014). These social determinants are what Marmot (2018) would call the “causes of the causes;” the factors that structure exclusion, social isolation, and disparity in participation.

This article aims to examine the impact of one of these “causes of causes: targeted violence and persistent victimization. We do this chiefly by exploring their role in structuring people with learning disabilities” experiences of communities and, in turn, their health and wellbeing. Community involvement, of people with learning disabilities, is now a key component of their support. One of the recommendations in the UK Transforming Care report (Department of Health, 2012) into the Winterbourne “scandal” was that adults with learning disabilities and “challenging behavior” should be integrated into and supported in their homes, near their families, and in communities.

The promotion of community participation and community inclusion has become central to policy responses shaping services for people with learning disabilities across the United Kingdom and beyond. For example, the Scottish Government’s “Same As You?” (2000)^4^4The Same As You (2000) Easy Read Consultation document can be found at https://www.gov.scot/publications/same-2000-2012-consultation-easy-read/ and “The Keys to Life” (2013 and 2018) emphasize the need to support people with learning disabilities to “live independently in the community wherever possible” (Ministerial Foreword) and that “everyone should have the opportunity to contribute to the communities in which they live, work and socialize” (2018, p. 2).^5^5The Keys to Life Strategy can be found at https://keystolife.info/strategy/ In England, “Building the Right Support” (2015, p. 5)^6^6Building the Right support information can be found at https://www.england.nhs.uk/learning-disabilities/natplan/ states that “we need to see people with a learning disability and autism as citizens with rights, who should expect to lead active lives in the community.” Despite significant policy recognition of the rights and wellbeing of people with learning disabilities, violent victimization, and community exclusion is commonplace (Power & Bartlett, 2018b).

By drawing on critiques of “community” (Bauman, 1997, 2013) and critical theories of “disrespect” and recognition (Honneth, 1996; Taylor, 1997), we propose a sociological approach to community, violence, and wellbeing. This approach allows us to move beyond a discussion wholly focused on causation and towards one that uncovers the layers of invisibility and complex relations that structure experiences from the perspective of people with learning disabilities themselves. By doing this, we locate violence and victimization as health and wellbeing concerns and seek to add a more comprehensive and holistic understanding of the social determinants of health.

## Review of the Literature

### Community, Belonging, and Respect

Community is a contested and dynamic concept, and while our intention here is not to define it, we want to examine how everyday experiences of a community might intersect with wellbeing and violence in the context of learning disability. Community has played a significant role in the history of learning disability in the United Kingdom, with most people with learning disabilities occupying marginal positions in society (Hall, 2005, 2010). “Community” is often understood to be a social good, to promote belonging and processes of interpersonal social relations that benefit individuals’ sense of wellbeing and safety. To have a “community” or “to be in a community” is, as Bauman argues, *perceived* to be inherently good (2013). For communities to *be* “good,” they must be inclusive, based on shared values, responsibilities, and “mutual care” (Bauman, 2013, pp. 149–50) and through “moral proximity,” shared concern and responsibility for others (Goodsell et al., 2014).

Community requires mutual recognition of rights and the recognition of personhood of all its members, as Honneth (1996) argues. For many people with learning disabilities, whose experiences are structured through exclusion and disrespect, moral proximity, shared responsibility, and care are often absent. To be treated well in communities, and to be afforded the “shared concern” that Goodsell et al. (2014) identify, respect must be afforded between persons; that is, to be given space for autonomy and afforded value (Honneth, 1996). Disrespect, for Honneth, or misrecognition “can inflict damage … the projection of an inferior or demeaning image on another can actually distort and oppress to the extent that that image is internalised” (Taylor, 1997, p. 36). Reeve (2020) and Thomas (1999) theorize similar concepts of misrecognition focused on how the ableist and disablist attitudes that underpin societies result in psycho-emotional disablism whereby disabled people face barriers to “being” and “doing” thus constraining their life chances and wellbeing.

It is only relatively recently that, in the United Kingdom, people with learning disabilities have moved from long-stay institutions or segregated provision into communities, and, for some, long-term institutionalized living is still prescient (Fish, 2017; Johnson, 1998) and this legacy of exclusion from everyday social relations have rendered them of less perceived social value and bearers of fewer rights than non-disabled people. People with learning disabilities often are limited to being in communities of shared identity, or “safe havens” (Power & Bartlett, 2018a) with other people with learning disabilities where they feel safer but may remain segregated (Hall, 2010, refer also to Bauman, 2001). Friendships are not easily made or maintained for people with learning disabilities, and this social isolation reinforces their marginal positions (Power, 2013) thus creating conditions for misrecognition and victimization. Exclusion is complex and is the result of systemic misrecognition and nonbelonging and resulting in individual victimization, and socio-spatial exclusion from neighborhoods and communities (Hall & Bates, 2019). As Power (2013) argues, a lasting and meaningful sense of belonging for people with learning disabilities “calls for a renewed debate … to ensure the processes, staff, and services are there to facilitate community living based on creating a sense of belonging” (Power, 2013, p. 72). However, and arguably, that sense of belonging is contingent upon “who is allowed to take part in the reflexive arguments that contribute to changes in society, who are excluded from these … and the effects that such inclusion and exclusion have on people’s sense of self” (May, 2011, p. 374). That is, the feeling of belonging is produced through being included in the policies, structures, and processes that form social life which people with learning disabilities have been, and continue to be, excluded from.

### Violence and Learning Disability

Violence plays a significant role in the lives of disabled people, and disabled people experience violence at disproportionately high levels compared to non-disabled people in the United Kingdom (Mikton et al., 2014). Violence has varying definitions; in this context defining violence in narrow terms is unhelpful (Hollomotz, 2012). Violence is complex and intersectional. In this article, we draw on, and extend, the World Health Organisation’s^7^7
https://www.who.int/violenceprevention/approach/definition/en/
 framework for violence with violence being:

the intentional use of physical force or power threatened or actual, against oneself, another person, or against a group or community, that either results in or has a high likelihood of resulting in injury, death, psychological harm, maldevelopment, or deprivation.

Similarly, the WHO world report on violence specifically locates violence as a public health issue. It includes neglect (and medical neglect) within its definition of “maldevelopment and deprivation” (Krug et al., 2002). This definition reflects, to some extent, feminist influences on understanding violence as more than physical threat or injury (Kelly, 2013). The WHO includes emotional, psychological, or symbolic abuse (hooks, 1997). A feminist approach to violence recognizes violence to be bound up with power and oppression (Morgan & Björkert, 2006). In a similar way to the oppressive outcomes of gendered power dynamics, disablist power dynamics operate to normalize, while simultaneously render invisible, the “violences of everyday life” (Kleinman, 2000, p. 227, cited in Morgan & Björkert, 2006).

Violence toward disabled people takes many forms and includes abuse and neglect, hate crime, targeted violence, bullying, and harassment as well as intimate partner, gender-based violence, and interpersonal violence (Balderston, 2013; Emerson & Roulstone, 2014; Mikton et al., 2014). As Quarmby (2008) highlights, there is an increasing awareness of disablist hate crimes and hate incidents that range from targeted name-calling, bullying, and harassment to physical abuse and murder. Violence can also take the form of neglect and indifference (Quarmby, 2008; Heslop & Hoghton, 2018). People with learning disabilities are considerably more likely to be subject to hate crime, harassment, or bullying than other disabled people. They experience higher levels of targeting and abuse in institutional care settings, schools, by friends and people known to them (“mate crime”), and by support workers than other disabled people (Thomas, 2011; Sin, 2014).

People with learning disabilities are made more vulnerable to violence due to “how oppressive social systems and processes facilitate conditions within which violence is more likely to occur” (Hollomotz, 2012, p. 47). Scholarship (Roulstone & Saqique, 2012) around disablist hate crimes focus on understanding why hate crimes might happen. These narratives do not necessarily locate hate crime within broader structures of violence that are present at all stages of the life course for people with learning disabilities (Chakraborti & Garland, 2012, refer also to Chakraborti, 2015). Further, they ignore the histories of exclusion from communities that have located people with learning disabilities as “good to mistreat” (Hughes, 2019).

Violence against people with learning disabilities is profoundly under-reported, and we know that there are comparatively low numbers in comparison to other protected groups (Macdonald et al., 2017). The 2020 Hate Crime in Scotland statistics^8^8The Hate Crime in Scotland 2019–2020 report can be found at www.copfs.gov.uk demonstrates that while disability hate crime figures are at their highest since legislation came into place in Scotland (with 387) charges, these figures are considerably lower than other protected characteristics. These low numbers are not evidence of low levels of violence but rather under-reporting and the normalization of violence against disabled people (Macdonald et al., 2017). As Chakraborti & Hardy (2017) outlines, disabled people and people with learning disabilities, specifically, may find it challenging to report targeted violence not only because of inaccessible reporting systems but also out of fear of institutional reprisals from the police, for example. People with learning disabilities are also concerned that they will not be seen as credible or they face barriers to reporting as the person “supporting” them may also be the perpetrator of violence (Sin, 2014). Support workers, residential staff, and family members can also disregard acts of violence and encourage people with learning disabilities to “ignore” them. Additionally, the everyday nature, and the dehumanizing implications, of the violence that structures the lives of people with learning disabilities produce a process of normalization whereby people with learning disabilities may accept violence as given and “normal” (Williams and Tregidga, 2014, refer also to Hydén, 2015).

### Connecting Violence to Wellbeing

Violence has consequences. It shapes societies and produces “social change” (Walby, 2013), it is inherently linked to oppression and, in turn, structures how people experience their communities (Hollomotz, 2012). The relationship between violence and health requires examination; Hughes et al. (2012, p. 3) write that “understanding the magnitude of violence against affected groups is the first step in the public health approach to violence prevention.” In understanding how violence and health intersect, we take health to be the “ability to be and do things that make up a minimally good, flourishing and non-humiliating life for a human being in the contemporary world” (Venkatapuram, 2013, p. 21) and that health is, in turn, intertwined in interpersonal relations. Health and wellbeing are shaped by biological factors, and impairment effects play a role in how wellbeing is experienced. However, wellbeing and health are matters of social justice; to have a “good life” people “need to be surrounded by a supportive environment” (Venkatapuram, 2013, p. 1) and as Sen (2002, p. 659, cited in Venkatapuram, 2013, p. 2) states:

In any discussion of social equity and justice, illness and health must figure as a major concern. I take that as my point of departure … and begin by noting that health equity cannot be but a central feature of the justice of social arrangements in general.

Figueiredo-Ferraz et al. (2015) found that workplace bullying, or “mobbing,” played a significant role in amplifying depressive symptoms in people with learning disabilities in Spain. Similarly, King et al. (2018) found, in an Australian study, that bullying accounted for 46% of the mental distress experienced by disabled teenagers. McNicholas et al. (2020) found that disabled children who experienced peer victimization, in school, felt that being disabled was the reason for victimization. Alhaboby et al. (2016, p. 1140) describe how online harassment and cyberbullying of disabled people had a significant impact on wellbeing, leading to “distress, anxiety, mood disturbances, deterioration of existing health conditions, and suicidal attempts….” While these experiences often had lasting consequences on physical and mental wellbeing, the research also found that good social networks (supportive families, friends, siblings, and others) often mediated the long-term impacts of bullying and violence. These foundational social and supportive networks are often missing in the lives of people with learning disabilities.

The preventable deaths of people with learning disabilities in institutional settings and community spaces, due to systemic victimization, are the most immediate indicators of the relationship between violence and wellbeing. As Walby (2013, p. 98) reminds us, “violence wrecks and shortens lives” and, we argue, does so disproportionately for people with learning disabilities. In the United Kingdom, policy responses to violence and victimization have focused on criminal justice, although the rates of prosecution of disability hate crime offenses remain low and public health responses have been less forthcoming (Hughes et al., 2012).

The literature, above, indicates a need to bring these three substantive areas together and that is the purpose of this article. While violence, health, and community have been explored in detail in previous research, the role that belonging plays in the health and wellbeing of people with learning disabilities has not.

## Methods

The data presented in this article were drawn from a project on people with learning disabilities’ experiences of violent victimization in Scotland. We undertook this research using critical feminist phenomenology to connect participants’ narratives, lived experiences, and the structural forces that shape them (Baird & Mitchell, 2014). While historically, people with learning disabilities have been excluded from such research (Walmsley, 2001), there has, recently, been an increase in inclusive research that positions people with learning disabilities as authoritative narrators of their own experiences, and we aimed to position participant narratives as meaningful, expert accounts of violence and inequality.

### Sample and Research Procedure

Data were collected using in-depth semi-structured interviews with 12 adults with learning disabilities and two focus groups with ten adults with learning disabilities across four cities in Scotland. The research project was designed in collaboration with people with learning disabilities and with participation from self-advocates. They helped to develop the design of the interview guides and the inclusive communication methods to ensure that we could approach this topic in a way that minimized harm to participants. Participants took part in the research from an open recruitment call. Participants, in some interviews, were supported by support workers where the participant specifically asked for them to be present. Where a supporter was present, they did not answer questions on behalf of participants and did not take part in the interview. Easy Read materials (Callus & Cauchi, 2020) were designed, in collaboration with people with learning disabilities, using picture symbols. Talking Mats (Murphy & Cameron, 2008) was available if participants felt this would be an accessible tool to support participation. Participants guided interview topics, and their narratives were transcribed verbatim to represent their experiences. We did not offer any incentives for participation in interviews or focus groups.

### Data Analysis

The data were analyzed using thematic analysis (Braun & Clarke, 2019) with an analytical focus on phenomenological interpretative methodological practices. Use of narrative analysis (Riessman, 1993) centered on the lived experiences, feelings, and authorities of participants themselves, how they constructed the stories of their experiences of violence and how these are culturally constituted (Morgan & Björkert, 2006). The interviews were transcribed in full and continuously read, reread, and compared so the data could be meaningfully grouped into themes. The generation of themes was undertaken inductively, focusing on topics that participants had identified as being important to them, but that also had shared meaning around central phenomenological experiences of violence and wellbeing (Braun & Clarke, 2019). The four key themes presented in the “Findings” section are: “community violence,” “impacts on wellbeing,” “invalidation,” and “belonging.” Data were initially manually coded and then subsequently coded using NVivo 12 (Bazeley & Jackson, 2013) qualitative data analysis software.

### Ethical Considerations

All participants were 18 years or over and have been given pseudonyms chosen by them. Similarly, all identifying information has been removed to ensure anonymity. Consent was negotiated at the beginning, throughout, and at the end of the research process. Participants did not want their specific ages to be presented in the findings and preferred that an age range was given instead. Where participant ages are noted in the findings, they are done so using a broad decade category such as “20s, 30s, or 40s,” for example. In the presentation of the findings of conversation between the researcher and participant, the researcher is referred to with the abbreviation “Ph” and the participant with the first initial of their pseudonym. Where there is a direct quote only from a participant their full pseudonym is used. Ethical approval was granted through the institutional ethics committee.

## Findings

### Violence in Community Settings

Being subjected to violence was part of people’s everyday experiences throughout the life course and was experienced in various community settings such as schools, participants’ homes, public transport, and in care settings. Participants described trajectories of harassment and bullying that started from a young age. Schools were often the first space where participants experienced violence:

R: That bullying was at school as a kid.

Ph: Do you feel that was to do with having a learning disability?

R: Oh, yes, just because I was being different, so yes.

(Richie 30s)

Childhood victimization was part of the everyday fabric of school life and, for many, extended throughout their adult life, as Gary told us:

Well, I was bullied in school. I still get bullied now from one of the people from the high school I was at for a short spell.

(Gary 50s)

Women reported experiences of sexual violence as children and adults. Angie (a woman in her fifties), for example, told us that she had been a victim of sexual abuse by male family members, and this continued when she was at school where she experienced sexual assault. This experience had long-term impacts on her emotional and mental health and wellbeing. Participants also reported violence in their homes and while in care. Annie talked about having been abused by her mother’s (male) partner, which led to her being placed in state care:

It was hard going because I got bullied, I got slagged [made fun of], I got my stuff flinged [thrown] out my windows an all that. Like it was really difficult being in care because I was the only one in care with a learning disability … people used to sit and call me a mongo every time my school bus used to pick me up and take me to school. I used to get called names, “Aha you’re a window licker” and stuff like that, it wasn’t nice, you know? It’s just what happens to people like us.

(Annie 20s)

These experiences were routine as Annie says, “It is just what happens to people like us.” Most participants felt socially isolated, with very few having been able to maintain friendships. Like above, the frequency of violence rendered it typical for many participants.

A key space where participants accessed the wider community was on buses, which played a central role in allowing them to navigate their towns and cities. Buses were the main form of transport to get to the hospital, college, shops, social clubs, and activist networks and community centers, to see friends and family, and to take part in various social activities. Buses were also the most cited location where people felt targeted. Gary spoke about an event that involved his girlfriend, who also had learning disabilities:

G: [Pause]. I know my partner was tied to a bus pole on the bus and they turned around and tried to say that she tied herself to the pole. She wouldn’t do that.

Ph: Who did that?

G: That was a couple of youths on the bus.

Ph: She must have been terrified.

G: Oh, she was. She ended up bursting her toggles [buttons on her coat] to get herself free, that’s the only way she could get herself free.

This experience was made worse for Gary, and his girlfriend with a learning disability, because no one around them tried to stop the incident or help her, including the bus driver:

Ph: Did the bus driver do anything?

G: No, he just sat in his cab.

Charlie (30s) also described his experiences of being harassed on buses. He told how he had been physically attacked by three women who followed him to a bus stop and then on and off three different buses.

Three girls pushed me up against a window on a bus, and it was a horrifying experience—it’s hurt me ever since. These girls no matter where I went, they followed me. They pushed me up against the window and called me all the names, called me a mongo and everything—that was enough to send me over the edge. I ended up taking myself up to my bed—I didn’t even get dressed, I didn’t even go out for four weeks, I didn’t even do anything.

Participants reported similar experiences in shops. Tracy, a woman in her thirties, described how, when she was shopping and was taking time counting out her money, a man behind her started to harass her. The man became increasingly angry, eventually shouting at her “I pay my taxes so that people like you can stay in institutions.”

Not only were these incidents publicly visible, but they were also ignored by others who witnessed them. These expressions of devaluing were epitomized by other people doing nothing. They legitimized the permissible dehumanization of people with learning disabilities. This permissibility indicates that shared moral value and moral proximity does not extend to them.

### “I Became Something Called Depressed:” Impacts on Wellbeing

Acts of violence were not only in themselves painful and destructive, but these events had profound consequences on participants’ long-term health and wellbeing. Andy described how the violence he had been subjected to had made him too scared to leave his home:

A: Well, where I used to live, I couldn’t go out, because they called me names all the time. And they threw stones at me.

Ph: What kind of names were they calling you?

A: Spazzie, mongo, brain-damaged, stupid.

Ph: How did it make you feel?

A: I couldn’t go out, and now I don’t go out now. Too worried.

(Andy 40s)

Participants who were targeted in their communities became isolated, depressed, and afraid to leave home. It affected their ability to participate in the community. We asked Charlie how these experiences might have impacted his wellbeing, and he said:

C: Like, you can’t go out. I’d sit in all the time and feel isolated and feel like you can’t go out, and they call you names. And you get stones thrown at you.

Ph: And what was that like, feeling isolated?

C: You couldn’t go out with your friends. You couldn’t meet anybody, and you couldn’t talk to people.

Gary told us about a tunnel near his house where people would harass him if he tried to walkthrough:

I did feel scared in the house, I felt trapped, that if we would go out, they would just say something, and they’d prevent us from getting past because they were in the tunnel but on both sides drinking away and you could hardly get by them. But I got by with support from one of my support staff who walked me through because I didn’t want to do it on my own.

Gary went on to talk about how these same young people broke into his house while he and his girlfriend were there. The break-in meant that Gary’s girlfriend became too scared to stay with him, and he was too frightened to go out to see her. The impacts, of violence, on participants’ emotional and mental health also meant that they were sometimes too depressed and anxious to leave their homes for fear of being targeted:

Ph: Can you tell me what kind of things they shouted at you and called you?

A: Just Mongol or spastic or laughing at me and throwing things at me.

Ph: What kind of things?

A: Food.

Ph: How did you feel when all of that happened?

A: That was scary. I was nervous, and I got depressed.

Ph: Were you scared to go out?

A: Yes, that’s why I sometimes like … I had like cognitive behaviour [therapy], and sometimes I don’t really want to go to my front door because of scared. I got depressed. I feel like I didn’t want to be here or anything like that.

Ph: What happened when you felt like that?

A: I self-harm myself.

(Angie, 50s)

What is perceived to be “low levels” of bullying, such as name-calling, kicking doors, or shouting significantly harmed participants. Participants’ sense of wellbeing, safety, and belonging were steadily eroded when these incidents took place and, in a small number of cases, resulted in participants self-harming or attempting suicide. Annie talked about the impact that persistent and targeted bullying had on her mental wellbeing when she was in care:

When I was 13, I started self-harming and I’ve still got wee marks there and stuff, and I used to do a lot of self-harming to try and let some pain out. When I was 16, I tried hanging myself, but I didn’t succeed but it was just because everybody was slagging me, bullying me for having a learning disability, and it was getting a bit too much for me.

(Annie, 20s)

Like Annie, Gary also spoke about suicide. When we asked him how being targeted made him feel, he said:

It makes it bad for my mental health certainly, because it made me feel a bit depressed. [Pause]. Made me feel I just want it all to stop, tended to feel like I wanted to take my own life, basically.

Participants, like Angie, said the only way they got the harassment and violence to stop was to move to a new house or move to another neighborhood. In many cases, participants had to rely on third parties for support. Fear of reoccurrence of violence taking place meant that many participants would go out only with support workers present, which reinforced the idea, to participants, that people with learning disabilities can be safe or navigate communities only with protection from others. Suzy, a support worker, reflected on her experiences with people with learning disabilities, who she supported, and who had been subject to hate crime:

From the discussions I’ve had with people I support, it’s the impact on mental health, and it’s quite significant, so the stress and the anxiety that comes with that. For some people, depression and its counterparts come into play as well, but I definitely think the anxiety and the frustration. People say that they don’t feel like going outside, so it’s like they’re trapped in their own homes … they kind of feel like the world outside is this really hostile place. The element of isolation is quite a big deal, and it has quite an impact on anybody’s health really, but people with learning disabilities, a lot of them don’t have the social life that other people do. Then you know if their mental health deteriorates then they might stop looking after themselves or their house as well, and then that can have an impact on hygiene as well and eating.

When services failed to respond to participants’ reporting of violence or targeted harassment, the onus fell on participants themselves to ameliorate the effects of violence, either by remaining in their homes or moving to new neighborhoods where, in some cases, violence re-emerged.

## “You’re Talking Rubbish:” Not Being Believed

The devaluing of people with learning disabilities emerged in myriad spaces and contexts. Not being believed, constructions of incredibility and rejections of stories of hate crime were frequent and pervasive. When violence was made visible by participants, to others, their reports were actively denied. Police were identified, by participants, as being key perpetrators of invalidation. Police were often felt to be ineffectual and did not always follow up incidents that were reported to them. Tom (70s) felt that the police might not believe him:

Ph: Do they [police] do something about it?

T: I don’t know. They just take a statement for it and make me sign it and then go away.

Ph: Do you feel like the police believe you?

T: Well, that’s a good question too?

Ph: Do you worry that they don’t?

T: Aye.

Participants’ narratives around not being believed were some of the most profound experiences of devaluing and misrecognition, particularly when this came from families. When we spoke to participants about the police, they often felt that police ignored their reports of harassment and hate incidents, or that police treated them as if they were not credible, so Greg told us:

G: I told my mum.

Ph: And what did your mum say?

G: Just ignore them. They’re just white trash. Just ignore them.

Ph: And did you ever feel like you would call the police about it, or anything?

G: We did call the police. They didn’t want to be involved.

Ph: They didn’t want to get involved?

G: No.

Ph: Right. And did they ever come out to your house, the police?

G: No, never.

This example, from Greg, evidences the complexities in people with learning disabilities reporting hate crimes and feeling like they’ll be taken seriously by the police. Annie returned to the experience of being in care and telling staff on numerous occasions that she was being targeted:

I tried speaking to the staff, and they were, they were like that, “no they would never do that,” so they didn’t believe me either that I was getting bullied. So, it wasn’t very nice being in care, it was quite a bad experience I had ‘cause nobody believed me I was getting bullied, and when I did try and tell somebody, they didn’t believe me.

Families, support workers, and friends were, at times, also complicit in rejecting participants’ reports of violence or hate crime.

### Belonging

Angie had lived in a community in which she had been repeatedly and systematically targeted. After being harassed for years, Angie finally moved to another area, in a bid to get the targeting to stop, and talked to me about her new neighbors:

Yes, the neighbours are very nice. I was away on holiday last year in October, no, this year, and [my support worker said] tell them you’re going away. I don’t like going to people’s door, so I didn’t tell them, and I came back, and I found out they were all worrying about me because I wasn’t there. That made me feel good and important.

While a story of belonging, the responsibility is placed on Angie to ameliorate the effects or likelihood of victimization herself while the structures tasked with doing so are difficult to access, ineffective, or uninterested. The meaningful participation, visibility of and recognition of the value of people with learning disabilities produced positive outcomes for wellbeing as Jamie told us:

J: I actually got to go to the Commonwealth Games. I was a volunteer!

Ph: That’s cool, how was that?

J: Amazing!! I loved that. I got to meet new people, and I got a uniform.

Ph: What was your favourite thing about it?

J: People looked at me nice. They smiled, and they thought I was important. It made me feel I was worth it to be there.

(Jamie, 30s)

Simple acts of inclusion, recognition from others had lasting impacts on self-worth and acted as powerful expressions of the importance of belonging in the lives of participants.

## Discussion

Literature on disability hate crime and violence rarely locate belonging and wellbeing as central to understanding the consequences of victimization or to being central to health. We have brought concepts of community, violence, and wellbeing together to show that violence and pervasive victimization (Gormely, 2017) impacts people with learning disabilities’ health and wellbeing. As such, these findings demonstrate that the lived experiences of people with learning disabilities are the fundamental lens through which everyday violence and belonging should be understood. Through an intersectional focus on community, wellbeing, and violence, we have explored how community has simultaneous possibilities for harm and mitigating the consequences of harm. The findings show that a community public health approach to victimization may contribute to addressing the unequal life chances of people with learning disabilities by building on what Marmot (2018) refers to as “the causes of the causes.” As the findings indicate, 4 of the 22 participants made references to suicide attempts and repeated suicidal thoughts as a result of persistent targeting and violence over the life course; participants had experienced a physical injury, emotional and psychological distress, self-harm, and paranoia. Violent victimization removed the “supportive environment” that people require for, what Venkatapuram refers to as, a “good life” free of humiliation or bad treatment (Venkatapuram, 2013, p. 1). As such, the findings reflect Venkatapuram’s call for a more holistic view of wellbeing that is intertwined with community and belonging.

Communities can play a crucial role in creating the conditions for belonging and participation to be possible (Power & Bartlett, 2018b). These conditions are what Honneth refers to as “respect” and “recognition” (1996). However, the ableist conditions of communities are what makes the dehumanization of people with learning disabilities possible as identified in the findings. Loneliness, social isolation, and nonparticipation, in and of themselves, have destructive effects on people’s health and wellbeing (Holt-Lundstad et al., 2015). Ableist communities create these conditions through what Thomas terms, psycho-emotional disablism (Thomas, 1999), a term she coined to examine the way that the multiple forms of discrimination and invalidation experienced by disabled people can impact on and undermine their psycho-emotional wellbeing. They act as barriers to being, preventing disabled people from being who and what they want to be, from having recognition (Reeve, 2020).

Psycho-emotional disablism can be both direct and indirect, and both are shown in the findings presented in this article. These can be seen through the denial of credibility when people with learning disabilities reported violence and being ignored by those who witnessed violence.

Not being believed by others was a key theme to have emerged in this research. The findings resonate with previous research showing that barriers to reporting hate crimes to the police are faced frequently by people with learning disabilities where “officers have been reported not to have taken notes or even interviewed persons with intellectual impairments because there is an assumption that those persons will not have the ability to effectively give evidence” (Macdonald et al., 2017). Acts of dismissal compound the impact of violence on the individual by confirming their difference and low social value. Dismissing people with learning disabilities as less credible also impacts on those who witness disablist violence as they can be “inclined to develop permissive attitudes that allow them to reduce moral disagreement and shed personal responsibility” (Radkiewicz & Korzeniowski, 2017, p. 3799). Witnesses are more likely to passively permit acts of violence against “others” (particularly disabled people) than to justify violence. This was reflected in the research findings; however, the literature does not indicate how being devalued impacts wellbeing. The study found that while people with learning disabilities were certainly devalued by others, that this also had profound implications for mental health and wellbeing. Previous research shows that people with learning disabilities feel less able to report violent victimization when it occurs, but also where violence begins to feel “normal.” This article has shown that to mitigate daily victimization, people with learning disabilities take measures themselves which can result in further isolation and distance from communities in which they are victimized.

Examples of belonging, in the research, show that building belonging and “mutual concern” in communities, between its members, and including people with learning disabilities, has the potential to challenge the consequences of exclusion through the creation of “welcoming communities” referred to by Power & Bartlett (2018a). Although hard to come by in this research, and crucially distinct from the feelings of precarious comfort or security felt by being accompanied or supported by a support worker, it was imperative that stories of belonging be present here. They offer a crucial illustration of the impact that recognition and “community” belonging can have for people with learning disabilities who so frequently have their personhood denied or negated both by those who actively harm them and those who stand by while violence is occurring. This evidences a need for infrastructures of support and care that cultivate “shared moral proximity” (Goodsell et al., 2014) and belonging.

Being valued in a community is a mode of belonging, of feeling welcome by others that share the community. As Yuval-Davis points out, belonging is an expression of feeling “at home” (2006) and, crucially, of feeling “safe” (Antonsich, 2010). This existential feeling of belonging has a phenomenological resonance, the expectation of acceptance, inclusion, and safety (Wiseman, 2019). This article has shown that violent victimization results, not only in the denial of belonging but the destruction of wellbeing. As identified, people with learning disabilities relied on support from others, who were not stigmatized, meaning that they could not go out on their own, were increasingly reliant on other people for security, and remained within their “safe havens” (Power & Bartlett, 2018a) where they felt less targeted. Fear of being targeted meant that participants led segregated lives.

This study emphasizes the need to prioritize focusing on communities, as they are experienced by those who are excluded from them. While the research provides important insights into the life experiences of people with learning disabilities this is a relatively small study and focuses mainly on those living in (semi)urban areas, further research is required to explore the experiences of those living in rural settings and the experiences of younger people with learning disabilities.

## Conclusion

In this article, we have explored the relationship between community, violence, and wellbeing. We have argued that improving the health and wellbeing of people with learning disabilities requires that everyday violent victimization be considered as a public health concern. While attitudinal change is undoubtedly needed, this achievement must be realized through transformative policy change at community and state levels. Transformative policy change requires that institutional disablism epitomized in police interactions with people with learning disabilities, for example, and the inadequate provision of accessible information for people with learning disabilities be taken seriously by local authorities and the state. People with learning disabilities are disproportionately at risk of violent victimization, not by virtue of having an impairment, but as a result of a historical devaluing of their personhood and identity. While a “doubling down” on vulnerability and the increased surveillance, or safeguarding of people with learning disabilities may further hide them from view and therefore increasing their exclusion, engaging public health and community solutions may produce the necessary conditions for belonging.

Making violence visible and understanding its consequences for health and wellbeing demands that we “see violence not as ‘exceptional’ or ‘unusual’ events … (but) processes that routinely and over time, deteriorate our mental and physical health” (Cooper and Whyte, 2017). If we want to tackle the systematic discrimination experienced by people with a learning disability and their inequalities in health and wellbeing, the roles violence and community play in constructing these disadvantages have to be addressed. This requires not only policy reform that better recognizes and fulfills the rights of people with learning disabilities but considerable social reform and funding to better support meaningful belonging in communities. Further research is required to explore people with learning disabilities’ experiences of belonging in diverse settings, including rural settings, and to focus on women with learning disabilities’ experiences of gender-based violence.

## Author Biographies

**Phillippa Wiseman** is a lecturer in social research methods at the University of Glasgow. She is also an affiliate of the Scottish Learning Disabilities Observatory at the University of Glasgow. Phillippa has written on embodied citizenship and experiences of toileting for young disabled people. She is interested in disabled people’s experiences of intersecting inequalities, critical theory, citizenship, violence gender inequality through feminist research methods.

**Nick Watson** is a professor of Disability Research and director of the Centre for Disability Research at the University of Glasgow. He has written on a range of disability issues including disability and technology, disability and identity, and disability theory. He is also Director of What Works Scotland, an ESRC and Scottish Government funded research program exploring public service reform in Scotland and is a co-investigator on the Children’s Neighbourhoods Scotland, a place-based approach to tackle child poverty.
